# Effects of Insect Consumption on Human Health: A Systematic Review of Human Studies

**DOI:** 10.3390/nu15143076

**Published:** 2023-07-08

**Authors:** Nair Cunha, Vanda Andrade, Paula Ruivo, Paula Pinto

**Affiliations:** 1 Escola Superior Agraria, Instituto Politécnico de Santarém, 2001-904 Santarém, Portugal; 120318002@esa.ipsantarem.pt (N.C.); vanda.andrade@esa.ipsantarem.pt (V.A.); paula.ruivo@esa.ipsantarem.pt (P.R.); 2 Life Quality Research Centre (CIEQV), IPSantarem/IPLeiria, 2040-413 Rio Maior, Portugal

**Keywords:** cricket, worm, amino acid absorption, protein synthesis, satiety, biomarkers, allergy

## Abstract

Insects have been consumed as food in diverse cultures worldwide, gaining recognition as a sustainable and nutritious food source. This systematic review aims to update information on the impact of insect consumption on human health based on human randomized controlled trials (RCTs) and allergenicity assessment studies. Following PRISMA guidelines, studies published in the last 10 years were analyzed. From one-thousand and sixty-three retrieved references, nine RCTs and five allergenicity studies were analyzed. Post-prandial amino acid levels increased after insect protein consumption. In comparison with other protein sources, insect protein showed no significant differences in the area under the curve (AUC) values for essential amino acids but tended to have lower peaks and peak later. In terms of muscle protein synthesis, there were no significant differences between insect protein and other protein sources. Glucose levels did not differ; however, insulin levels were lower after the consumption of insect-based products. The effects on inflammatory markers and microbiota composition were inconclusive and the studies did not show significant effects on appetite regulation. Allergenicity assessments revealed a sensitisation and cross-reactivity between insect allergens and known allergens. A partial reduction of cross-allergenicity was observed via thermal processing. Insect protein is an adequate protein source with promising health benefits; however, further research is needed to fully understand its potential and optimise its inclusion into the human diet.

## 1. Introduction

Edible insects are considered a valuable source of highly nutritious food [1]. Eating insects is a common practice in several countries, such as Africa, Asia, Australia, Oceania, and Latin America [2]. In these countries, insects are an important source of nutrients for human diets and are also recognized for their medicinal properties [3]. There are more than 2000 species of insects that can be eaten by humans. Beetles, caterpillars, bees, wasps, ants, grasshoppers, locusts, crickets, true bugs, dragonflies, termites, flies, and cockroaches are the most consumed worldwide [4]. Insects can provide a variety of nutrients, such as high-quality protein, essential amino acids, mono- and poly-unsaturated fats, vitamins, and minerals [1]. The nutrient composition of insects can vary significantly depending on the species; however, protein and fat contents are the ones that appear in major quantities [5]. Furthermore, edible insects constitute an environmentally friendly source of food production as they require less feed, less water, and less land use; they also emit lower greenhouse gases compared to traditional animal protein sources [6]. This may be due to their high feed conversion ratio, high fecundity rates, and short life cycles [7,8,9]. As the Food and Agriculture Organization of the United Nations (FAO) reported [10], edible insects are an underutilized resource with great potential to be an innovative food that can offer numerous benefits to humans. Thus, insects can present a healthy, nutritious, and sustainable food choice for consumers.

This has led to interest from Western countries in insects like crickets and mealworms for human consumption, specifically the cricket *Acheta domesticus* and the mealworm *Tenebrio molitor*, as these are the species with greater potential to be used as human food [11]. However, the level of acceptance regarding edible insects is still low among Western consumers when compared to other alternative food sources like plant-based foods, mainly due to neophobia and repulse factors [12]. The food industry and food scientists are working to foster an acceptance of edible insects among consumers by conveying the message that insects are healthy and nutritious food sources with environmental benefits [13]. In Europe, edible insects as an emerging food source can be framed in the category of novel foods and novel food ingredients, which is regulated by the Regulation (EC) No 258/97 of the European Parliament and of the Council of 27 January 1997.

Concerning medicinal properties, insects have been employed in some countries as a nutraceutical food for a long time [14]. For example, in Nigeria, crickets (*Brachytrupes membranaceus*) are used as a food source to promote mental development and pre- and post-natal care [15]. In Asia, the Chinese beetle (*Ulomoides dermestoides*) is often used as an alternative form of treatment for diseases, such as asthma, arthritis, and tuberculosis [14]. In Brazil, the same species is used as a stimulant and to treat eye irritation and rheumatism [14]. Some species of cockroaches and ants are used to treat asthma and are consumed in the form of tea in different countries, such as Brazil and India [14]. Indigenous knowledge of medicinal compounds from natural sources can be a valuable tool in bioprospecting for pharmaceutical compounds. So far, most studies have been carried out in vitro or using animal models. Results show that edible insects may provide gastrointestinal protection, antioxidant and anti-inflammatory activity, antibacterial activity, immunomodulatory effects, blood glucose and lipid regulation, hypotensive effects, and a reduced risk of cardiovascular disease [1,12,16].

Despite the interest in and importance of edible insects in the human diet, there are some concerns about food safety issues since, like any other food of animal or plant origin, they may also contain exogenous and endogenous risk factors. There is still little knowledge on this subject [11,17]. Potential food health hazards from insects are categorized into biological, chemical, and allergy hazards. It is known that the occurrence and concentration of possible contaminants are strongly influenced by the management conditions, insect species, harvesting age, and type of feed used throughout their production process [17,18]. Food allergy (FA) is a major health concern in Western society, with a prevalence of around 3 to 4% in the general population and symptoms which may range from an oral allergy to extremely severe conditions, such as anaphylactic shock [19]. In some countries where insect consumption is common, studies have shown that the prevalence of allergic reactions to insects, and even death, is considerably high. For example, in North-Eastern Thailand, a study involving 2500 participants reported that 14.7% of them showed multiple symptoms of allergies after insect consumption [20]. In China, of all of the allergic causes for anaphylactic shock and fatalities in the collected Chinese literature, from 1980 to 2007, 14% were attributed to locust and grasshopper ingestion [21]. In addition to direct sensitization, a crucial aspect of insect allergies that needs to be elucidated is the immunoglobulin E (IgE) cross-reactivity between insects, crustaceous, and house dust mite (HDM) allergens, generally known as pan-allergens [18,20]. 

In view of the possibility of insect farming and commercialization in Europe, the European Food Safety Authority (EFSA) has requested scientific risk assessments on the use of insects as food, with a particular focus on allergenicity. This has confirmed the need to implement measures to control every step of the production chain in this new sector, as well as the importance of continuously evaluating and making public the potential effects on human health of consuming this new source of food, edible insects [17,18].

Much research has been performed on insect nutritional value and the development of insect-based food ingredients or products. Recently, more researchers have focused on the potential impacts of insect consumption on human health. Most existent reviews are based on in vitro or in vivo animal models, as well as a few in vivo human studies, mainly addressing children’s malnutrition. Thus, this systematic review proposes to update the information regarding the effects of insect consumption on the health of human adults, using randomized controlled studies and allergenicity assessment studies to evaluate the cross-reactivity between edible insects and other common allergies.

## 2. Materials and Methods

### 2.1. Search Strategy and Eligibility Criteria

This systematic review was conducted in accordance with the 2020 Preferred Reporting Items for Systematic Reviews and Meta-Analyses (PRISMA) guidelines [22]. Searches were performed during August 2022 in Pubmed, Web of Science, and Science Direct, specifying articles from the last 10 years (2012–2022), with the following keywords: (i) “edible insects” AND “health”, All Fields; (ii) “Gryllus OR Gryllodes OR Acheta OR cricket OR beetle OR Tenebrio OR worm OR Alphitobius” AND “health”, Title/Abstract. Only articles written in a European language were included. The screening was performed independently by three researchers, with any non-consensus discussed among the three to reach a final decision. The screening was primarily based on the title and abstract, followed by an analysis of the full paper when the available information was too insufficient to reach a decision on its acceptance or rejection and the reasons for rejection. The inclusion criteria were as follows: studies with edible insects, in vivo studies with humans measuring health-related outcomes, and adults. The exclusion criteria were: books; editorials; reviews; no edible insects; studies with edible insects that are not related to human health, such as consumer acceptance studies, insect utilization as feed, insect farming, insect composition, technology or development of laboratory methods, and studies focused on the physiology, biodiversity, or ecology related issues of insects; in vitro studies, except those that involved human sera for allergenicity assessment; animal studies; and human studies with children. The reference list and citations of eligible manuscripts were manually checked for additional relevant studies. On the 24 January 2023, another search was performed to retrieve any papers published between the 16 August and the 31 December 2022 regarding human intervention studies, with no papers retrieved on the subject.

### 2.2. Data Extraction and Assessment of Risk of Bias

Data were extracted independently by two researchers to an Excel template previously defined by the researchers involved in this study. Doubts were cross-checked by a third reviewer. Extracted data included: (i) study characteristics: type of study (crossover, parallel, randomized or not, controlled or not), number of test participants, number of control participants, type of control, insect product (test), administration of test and control, and duration of intervention; (ii) characteristics of participants: country where the study took place, age, gender, and healthy or with diagnosed disease; and (iii) outcomes: for each analyzed parameter, data collection method and conditions, and observed result (significant or no significant differences between test and control, as well as the direction of the effect). A standardized form was constructed based on the Cochrane Collaboration’s tool for assessing the risk of bias for human studies [23], including the following domains: (1) risk of bias arising from the randomization process (generation of the random allocation sequence and explanation of allocation concealment); (2) risk of bias due to deviations from the intended intervention (blinding of participants, blinding of investigators, and methods for checking compliance); (3) risk of bias due to any potential carryover effects before starting the following intervention in crossover studies (washout time appropriate for the disappearance of carryover effects); (4) risk of bias due to any missing outcome data (flow of participants described and existence of outcome data for all participants that finished the protocol); and (5) risk of bias arising from the outcome measurement (appropriate method to measure outcome, any potential influence by knowledge of intervention or mismatch of test and control products). 

Overall evaluation: low risk of bias when all assessed domains are low risk; some concerns if any domain was assessed as having some concerns AND no domain was assessed as being high risk; high risk of bias when at least one domain was assessed as being high risk OR multiple domains were assessed as having some concerns [23] (Supplementary Table S1).

## 3. Results 

### 3.1. Selected Studies

**Figure 1:**
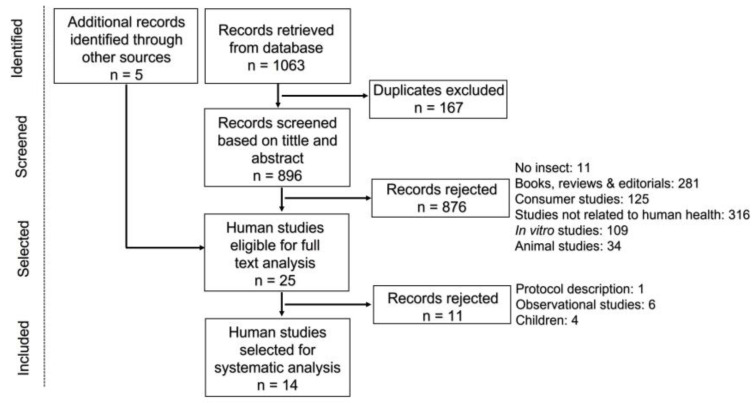
Flowchart of study selection.

A total of 1063 references were retrieved via the database searches. After the removal of duplicates, 896 records were screened independently by three authors, based on their titles and abstracts. The eligibility criteria were met by 20 studies. Manually checked references retrieved five more eligible studies. In the end, 25 articles were selected for full-paper analysis, from which 11 were rejected (Figure 1). Thus, 14 studies were included in the systematic analysis. Nine studies were randomized controlled trials (RCT), with the consumption of insect-based meals as the test product [24,25,26,27,28,29,30,31,32] (Table 1). The other five studies addressed allergenicity (Table 2): one was a patient study case [33], one was an epidemiological study that assessed exposure to insect allergens by skin pricks [34], and three were cross-reactivity studies involving patients’ sera exposure to extracts containing insects’ allergens [35,36,37].

### 3.2. Oral Interventions with Randomized Controlled Trials (RCTs)

#### 3.2.1. Characteristics of the RCTs

The nine RCT studies were divided according to the primary outcomes studied: iron absorption [31], amino acid absorption and muscle protein synthesis or strength [25,26,28], amino acid absorption and appetite regulation [29,30,32], microbiota [24], and disease treatment, specifically, Chronic Obstructive Pulmonary Disease (CPOD) [27] (Table 1). Six studies presented a high risk of bias, one had some concerns, and two studies had a low risk of bias (Table S1). Except for the COPD study, all studies were performed with young healthy adults (mean ages about 23 to 25 years). The study on iron absorption included only women, the trials focused on amino acid absorption and muscle protein synthesis or strength included only males, and the other four studies included both men and women. Most of the RCTs were acute studies and only three were chronic (2 weeks, 8 weeks, and 3 months) (Table 1). The test products included protein or flour derived from crickets (*Acheta domesticus*, *Gryllodes sigillatus*), lesser mealworms (*Alphitobius diaperinus*), mealworms (*Tenebrio molitor*), and silkworms (*Bombyx mori, Bombyx Batrycatus*). 

#### 3.2.2. Iron Absorption

Results on iron absorption were different for high- (porridge with unrefined maize flour) and low-phytate meals (porridge with refined maize flour). After the consumption of low-phytate meals, the iron absorption was significantly lower for test meals with crickets compared to the placebo meals; meanwhile, for the high-phytate meals, no significant differences were observed between the test meals with cricket and the placebo. Nevertheless, no significant differences in the participants’ plasma hemoglobin and serum ferritin were observed between the test meals with cricket and placebo meals, irrespective of the meal-phytate content [31] (Table 1).

#### 3.2.3. Amino Acid Absorption and Muscle Protein Synthesis

Three studies presented postprandial amino acid levels after the ingestion of 25 to 30 g of insect protein-based test product compared to other animal proteins (milk, whey, or beef) or plant protein (soy). Compared to the placebo, the consumption of protein increased the levels of amino acids, independently of the protein source [25]. When comparing insect sources to other protein sources, two studies used lesser worms, reporting lower peak values of leucine, branched-chain amino acids (BCAA), essential amino acids (EAA), non-essential amino acids (NEAA), and total amino acid (TAA); however, there were no significant differences in the area under the curve (AUC) values of these amino acids after insect protein ingestion when compared to milk protein [28]. and Lower AUC values were observed for leucine, BCAA, and EAA after insect protein ingestion compared to whey protein [25]. Another study compared the ingestion of 25 g of protein derived from cricket or beef protein [29], showing different results in the absorption of amino acids. In fact, significantly higher AUC values of leucine, BCAA, and EAA were observed for the insect-based product, compared to beef protein; meanwhile, lower values were observed for NEAA and TAA. No significant differences were observed in the amino acid levels after insect protein ingestion compared to a vegetable-protein-based product [25].

Notably, amino acid plasma levels peaked later after insect protein consumption compared to milk, whey, soy, and beef proteins [25,28,29].

Studies determining the effects of insect protein consumption on muscle protein synthesis reported no significant differences in protein synthesis rate, body composition, or muscle strength compared to an isocaloric bar without insect protein [26,28]. 

#### 3.2.4. Biomarkers of Metabolic Diseases

The plasma levels of glucose and insulin were determined in four studies [24,25,28,29]. No significant differences were observed for glucose levels after the ingestion of insects compared to other protein sources or an isocaloric placebo. However, results for insulin were different; while insulin levels after insect protein and milk protein ingestion showed no significant differences [28], a lower level of insulin was observed for insect-based products compared to those comprising soy, whey [25], and beef protein [29]. Regarding biomarkers of inflammation, Stull et al. (2018) observed a significantly lower level of tumor necrosis factor (TNF-α) in plasma after 14 days of consuming a test meal with cricket powder compared to an isocaloric placebo meal [24]. Other cytokines and chemokines measured showed no significant differences, as well as sIgA, a marker of mucosal immunity [24].

#### 3.2.5. Appetite Regulation

Regarding appetite regulation, no significant differences were observed regarding the desire to eat and the prospective food consumption between insect protein products and other protein sources, such as beef protein [29] or almond protein [30]. A higher sensation of indigestion was reported for insect products in one study [30]; however, another study did not observe any significant differences in feelings related to digestive health between participants that consumed insect products or the placebo [24].

One study compared different insects and quantities on hunger and satiety, reporting lower hunger after consuming products containing higher quantities of cricket and lesser worm [32]. Significant differences were observed between men and women in this study. 

#### 3.2.6. Microbiota

The study conducted by Stull et al. (2018) reported some contradictory results regarding insect consumption and microbiota probiotics, with results showing decreased levels of *Lactobacillus spp*. and increased levels of *Bifidobacterium*. Regarding microbial metabolism, this study observed a decrease in acetate and propionate short-chain fatty acid (SCFA) synthesis by microbiota in participants who consumed the meal with cricket [24].

#### 3.2.7. Disease Treatment

Finally, one study tested the impact of supplementing the routine medication of chronic obstructive pulmonary disease (COPD) patients with a combination of compound Caoshi silkworm (*Bombyx Batryticatus*) granules and astragalus root, both widely used dietary supplements in traditional Chinese medicine, for three months [27]. Although no significant differences were observed in the patients’ pulmonary functions between those taking the test and placebo, the scores of respiratory symptoms, activity, and impact improved in patients that supplemented the routine medication with the granules.

### 3.3. Allergenicity Assessment Studies

#### 3.3.1. Case Study

A case report conducted in France [33] (Table 2), described a severe food anaphylaxis being induced by the mealworm (*Tenebrio molitor*) in a 31-year-old man allergic to HDM but not to crustaceans, who consumed one cooked larva. Prick-tests and a serum proteomic analysis of the subject allowed us to identify the *Tenebrio molitor* proteins to which he was sensitized, most precisely, hexamerin, tropomyosin, α-amylase (previously identified as an allergen in mealworm with a structural homology with HDM), and larval cuticle proteins A1A and A2B (both known mealworm allergens) [33].

#### 3.3.2. Epidemiological Studies

In Zimbabwe, Ndlovu et al. (2021) performed a RCT to evaluate the clinical significance of allergens from a popular indigenous edible insect, the mopane worm (*Imbrasia belina*), in a vulnerable rural community occupationally exposed by harvesting [34] (Table 2). After responding to a questionnaire, the patients were exposed to an in-house preparation of the mopane worm inhalant allergen extract by a skin prick. Allergen sensitization was assessed by skin prick test patterns, measurements of lung function by spirometry, and fractional exhaled nitric oxide levels, which are markers of allergic airway inflammation. Respiratory health symptoms were detected among participants sensitized to the mopane worm and the results showed that 50% of the participants were sensitized to the mopane worm [34]. Additionally, mopane worm harvesting seems not to be the only determinant for mopane worm sensitization as approximately equal proportions of harvesters were found in sensitized or non-sensitized groups, along with the observation that 50% of the sensitized subjects were not harvesters. Additional data from this study showed a prevalence ranging from 22 to 72% for other allergens, which included cockroaches, mosquitoes, and HDM [34].

#### 3.3.3. Cross-Reactivity Studies

Three in vitro cross-reactivity studies involving patients’ sera exposure to insects’ allergen extracts were analyzed (Table 2). One of these studies was performed in Japan and aimed to assess a *Gryllus bimaculatus* (cricket)-induced allergy in shrimp-allergic subjects, using as a control group the sera of subjects without a shrimp allergy [36]. The identification of allergenic proteins in *Gryllus* and shrimp was followed by the estimation of allergen-specific IgE levels for shrimp and *Gryllus* in the sera of the subjects after the exposure of the samples to the insects’ protein extracts. Cross-allergenicity was found to occur between the cricket and shrimp, with the binding of shrimp-specific IgE inhibited by the *Gryllus* allergen in a dose-dependent way. A protein of approximately 40 kDa reacted with the positive, but not with the negative, sera patients for shrimp-specific IgE and was identified as a high molecular weight (HMW) tropomyosin [36]. Indeed, HMW tropomyosin was concluded to be the major allergen in shrimp and *Gryllus* and a cross-reactive allergen between both species. As proposed by the authors, shrimp-allergic subjects seemed to be at a higher risk of developing an allergy to cricket when considering that the identified cricket allergen had the potential to induce an allergic reaction in individuals with a crustacean allergy. These results led to recommendations of considering allergy risk by shrimp-specific IgE levels before the consumption of a cricket meal [36]. 

A similar study was performed in the Netherlands (Table 2); however, it involved another edible insect, the yellow mealworm (*Tenebrio molitor*) [35]. Again, the authors’ working hypothesis was that in subjects allergic to crustaceans and HDM, cross-reactivity with the yellow mealworm allergens could occur. The sera of these patients were exposed to different yellow mealworm protein fractions and the results were compared with the ones from the subjects who were allergic to grass pollen, peanuts, fish, or eggs and/or milk but not to crustaceans or HDMs (control group). IgE from HDM- and crustacean-allergic patients cross-reacted with yellow mealworm proteins and functional cross-reactivity was posteriorly proven by the induction of basophil activation. Two known pan-allergens, tropomyosin and arginine kinase, were identified as major cross-reactive allergens in the yellow mealworm. Regarding tropomyosin, the authors mentioned that while it is a major crustacean allergen, the protein is a minor allergen in HDMs. In fact, the dominant HDM allergens are Der p 1 and Der p 2, which are recognized by over 90% of HDM-allergic patients; meanwhile, only 5–15% of patients recognize the allergen Der p 10. However, patients with both a HDM and crustacean allergy express higher levels of IgE with Der p 10 than with the major HDM allergen. Therefore, patients allergic to crustaceans/HDMs Der p 10 may also experience an allergic reaction when consuming products containing yellow mealworm protein; it is imperative to test those subjects before such a meal consumption [35].

The third study was performed in Italy and involved several edible insect species: the buffalo worm (*Alphitobius diaperinus*), the mealworm larvae (*Tenebrio molitor*), the cricket (*Gryllodes sigillatus*), the grasshopper (*Locusta migratoria*), and the silkworm larvae (*Bombyx mori*) [37]. This study differed from the aforementioned works since the authors’ aim was to assess the effect of thermal processing on the allergenicity potential of such food sources. The insects’ protein profiles were determined after each thermal treatment (boiling or frying); further, the sera of patients allergic to HDMs, shrimp, or mealworms and the sera of subjects not allergic to either shrimp or HDMs (control subjects) were challenged for immunorecognition with thermal processed or raw protein extracts of the five insect species. Of 17 subjects, 1% of HDM-allergic patients and 87% of shrimp-allergic patients recognized at least one insect protein extract. Again, tropomyosin was mentioned as playing an important role as a cross-allergen for HDM- and shrimp-allergic patients; additionally, larval cuticle proteins seemed to play a major role in the cross-reactivity of patients primarily sensitized to mealworms. Most importantly, the obtained results showed that different proteins, some of them found to be thermostable, were involved in cross-sensitization and the effect of thermal treatment on the IgE cross-recognition of the allergens was protein-, species-, and treatment-specific. The results showed that thermal processing partially reduces cross-allergenicity; however, HDM-, shrimp-, and mealworm-allergic patients were advised to be cautious when consuming insects [37].

## 4. Discussion

Due to the increase in population, the pressure of high protein demand, and the concerns related to the sustainability of our planet, a trend towards the diversification of protein sources has emerged in recent years. Many edible insects are protein sources of high quality due to their elevated content of protein and essential amino acids and are also healthy sources of unsaturated fats, vitamins, and minerals [1]. Recent reviews of in vivo studies on the effect of edible insects on human health focused on animal studies, showing potential benefits, such as gastrointestinal health, anti-inflammatory properties, increased immune response, anti-dyslipidemia, anti-obesity, anti-diabetic, and anti-hypertensive properties [1,38,39]. Some human studies were also reviewed, mostly focusing on the potential utilization of insects to tackle malnutrition in infants and children [38,39]. Although insect consumption has a long tradition in Latin America, Africa, and Asia, only recently have insect farming and consumption reached the Western world [40]; thus, not many intervention studies have been conducted with human adults. To our knowledge, this paper is the first systematic review of human randomized controlled trials (RCTs) involving adults focusing on the health effects of insect consumption. Nine RCTs were eligible for analysis, with a total of 251 participants, mostly healthy young adults. Primary outcomes included iron absorption, amino acid absorption, muscle protein synthesis, satiety, some plasma metabolic and inflammatory markers, and microbiota analysis. Knowing the importance of allergy-related issues, studies with human allergic reactions and studies with human sera testing allergy cross-reactivity between several edible insect species and other allergens, such as shrimp and HDMs, have also been selected for analysis.

### 4.1. Beneficial Health Effects

#### 4.1.1. Nutrient Absorption

Insects’ iron content may be of particular importance to tackling iron deficiency in regions with elevated food insecurity. One study in this review addressed iron absorption in iron-depleted young adult females, showing no significant differences in the participants’ plasma hemoglobin and serum ferritin after the ingestion of a meal with cricket powder and after the ingestion of a placebo meal [31]. A few other human studies on the effect of insect consumption on iron status have been undertaken with infants and children, showing contradictory results: there were either no effects on iron status, or a decrease in plasma ferritin, or an increase in hemoglobin and a decrease in iron-deficiency anemia [38]. These results may arise from the different fiber contents in different insect species as this can impact iron absorption [31].

Regarding amino acid absorption, consuming 25 to 30 g of protein increased postprandial amino acid levels compared to the placebo, regardless of whether the protein source was from an insect, whey, or soy [25]. When comparing protein from insects to that of other protein sources, different amino acid absorption patterns were found. Cricket-based products had significantly higher absorption AUC levels for leucine, branched-chain amino acids (BCAA), and essential amino acids (EAA) compared to beef protein [29]. Lesser-mealworm-based products had similar values to milk protein [28] and soy [25] but significantly lower values compared to whey [25]. Notably, amino acid plasma levels peaked later after the consumption of insect protein compared to after the consumption of milk, whey, soy, and beef proteins. 

There is evidence that about 55% of dietary protein-derived amino acids will be released in circulation during the 5 h post-prandial period, making amino acids available as precursors for de novo muscle protein synthesis [41]. On the other hand, it is long known that the digestion rate of the consumed protein affects the incorporation of dietary protein-derived amino acids into skeletal muscle protein. Rapidly digestible proteins (such as whey) may stimulate protein synthesis to a greater extent and for a shorter period; whereas, slowly digestible proteins (such as casein) may stimulate protein synthesis for a longer time [42]. However, this effect may be different depending on the age [43]. The studies analyzed in this systematic review used the same matrix for the delivery of insect-based protein and other protein sources, as well as using the same quantity of protein. Thus, food matrix and protein dose can be excluded when attempting to explain the observed differences in the overall absorption and timing of the peak levels for the insect-based protein compared to the other protein sources. It is more likely that the observed effects are due to a slower digestion of the insect protein and, consequently, a slower release of the derived amino acids. In fact, it has been suggested that the presence of chitin in insects (a fibrous substance found in the exoskeleton of insects) may delay digestion, similarly to what is observed for high-fiber meals [31,44]. 

There is a complex interplay between dietary factors, protein digestion, and the subsequent release of exogenous amino acids into the circulation which ultimately affects protein synthesis in skeletal muscle tissue. Beyond the quantity of the protein and the digestion rate, the effectiveness of a protein in stimulating postprandial muscle protein synthesis highly depends on amino acid composition, particularly, leucine content [45,46]. It has been shown that graded intakes of essential amino acids up to 10 g (equivalent to ~25 g of a high-quality protein) stimulate myofibrillar protein synthesis rates in a dose-dependent manner [47]. Results from the present analysis show that the AUC for EAA and leucine after insect protein ingestion is higher than it is after beef protein ingestion and similar to what it is after milk and soy, suggesting that this protein source is an adequate protein alternative to promote muscle protein synthesis.

The co-ingestion of protein and carbohydrates or fat impacts amino acid release into the circulation, as well as food processing. For example, protein hydrolysates and minced meat result in an accelerated release compared to intact proteins or beef steak [46]. Thus, as with other protein sources, it is likely that the release of amino acids from insect protein would depend on various factors, such as the form of the protein (whole insect, insect flour, or insect protein isolate), the processing methods, and the presence of other ingredients in the meal or supplement. In general, whole insects or insect flour may have a slower release of amino acids due to the presence of indigestible components, such as chitin and chitosan, requiring more time to break down and release the amino acids. On the other hand, insect protein isolates that have been processed to remove non-protein components may have a faster release, similar to that of other protein isolates.

#### 4.1.2. Satiety

From a nutritional point of view, insects have significant protein content, varying from 20 to 76% of dry matter depending on the type and development stage of the insect; this is even the case when compared with meat, which ranks among one of the protein-rich foods, with percentages varying extensively in different types of meats and exhibiting an average value of about 22% [48,49].

Many studies have investigated the effects of proteins on satiety and most have found that at sufficiently high levels, proteins have a stronger effect than equivalent quantities of energy from either carbohydrates or fats [50]. A number of mechanisms have been proposed, including a higher thermogenic effect of dietary proteins and postabsorptive small intestinal gluconeogenesis [51]. Accordingly, this review includes a study in which different lower hunger values were reported after the consumption of crickets and lesser worms [32]. This may be explained by the higher contents of protein in these insect species. Kim et al. (2019) compared several insects’ protein contents; two cricket species *Gryllus bimaculatus* and *Teleogryllus emma* had the highest (58.32%) and the 3rd highest protein content of all the analyzed species (55.65%), respectively [52]. In another study, the protein content of lesser mealworm powder was reported to be 58.40%, which is even higher than that found in crickets [53]. 

In regards to appetite regulation, it is known that diets containing higher dietary protein generally reduce postprandial hunger and increase postprandial satiety, which is attributable to induced changes in hormone levels associated with appetite regulation, such as ghrelin and peptide YY [54]. However, no significant differences were observed in terms of the desire to eat and the prospective food consumption between insect protein products and other protein sources, such as beef protein [29] or almond protein [30]. We consider it a hypothesis that the amino acid content of the insect’s proteins did not have beneficial effects on appetite regulation in this study. Amino acids deriving from the digestion of proteins present in our diet may be chemo-sensed, not only at the peripheral level by enteroendocrine cells but also at the central level by hypothalamic neurons; single or mixed amino acids can influence the secretion of some gastrointestinal peptides and, consequentially, regulate appetite. It was in such a context that a study was performed by Rigamonti et al. (2020) to evaluate the effects of an amino acid mix (L-arginine + L-leucine + L-glutamine + L-tryptophan) on the secretion of some gastrointestinal peptides (i.e., ghrelin and glucagon-like peptide type-1, GLP-1), glucometabolic homeostasis (i.e., glucose, insulin, and glucagon), and appetite (hunger/satiety scored by a visual analog scale, VAS) in obese adolescents [55]. Indeed, L-arginine, L-leucine, L-glutamine, and L-tryptophan, administered to obese adolescents with a fixed-dose meal, were capable of evoking an anorexigenic response. In regards to edible insects, after analyzing the amino acid profiles of the Bombay locust, scarab beetle, house cricket, and mulberry silkworm, in Thailand, leucine was shown to be the amino acid with the highest concentrations (1.70 g to 4.08 g/100 g) of all the insect samples; however, tryptophan had the lowest values (0.23 g to 0.72 g/100 g) among the essential amino acids [56]. On the other hand, house crickets contained the highest amount of leucine while the mulberry silkworm had the highest amounts of tryptophan among all the insect samples; meanwhile, glutamic acid was of the highest concentration in the house cricket purchased from the street (3.84 g/100 g) [56]. These results evidence great variability in the amino acid content among insect species. Following the rationale of the mentioned authors, who claimed that more information is needed on the quality of insect proteins to be able to fully assess their value in comparison to other food proteins, we consider that our hypothesis should be consubstantiated with more data on the amino acid content of more insect species and on their associations with appetite regulation.

#### 4.1.3. Metabolic and Inflammatory Biomarkers

Secondary outcomes of the studies analyzed in this review included postprandial plasma glucose and insulin levels. Three studies reported no significant differences in glucose levels after insect protein powder consumption (lesser mealworm or cricket) compared to milk protein, beef protein [28], or a placebo [24]. However, the insulin levels were lower after insect protein consumption compared to other protein sources [25,29]. This suggests that insect consumption may have an impact on the insulin response. Studies with rat models have shown that insect consumption may have antidiabetic effects. For example, the administration of cricket powder to a streptozotocin (STZ)-induced rat model of type-1 diabetes significantly improved its results in the glucose tolerance test and insulin tolerance test [57]. A recent study has reported that 8% cricket powder supplementation to rats fed on a high-fat-high-fructose diet improved insulin resistance, as measured by the HOMA index [58]. These findings support the notion that insect consumption may have beneficial effects on glucose regulation and insulin sensitivity, indicating its potential as a dietary intervention for managing diabetes or improving metabolic health. 

Inflammation is an important aspect of the pathophysiology of major chronic diseases, such as cardiovascular disease, type-2 diabetes mellitus, Alzheimer’s disease, and many types of cancer, with nutrition being a modifiable risk factor for these pathologies [59]. Thus, many dietary intervention studies have been conducted to assess the impact of consuming foods on a variety of inflammatory diseases. Even so, due to the complexity of both inflammation and nutrition science, many details remain unknown about the broader links between different dietary factors, including the consumption of specific foods and the elevated markers of immune activation seen in chronic disease states [60]. 

Edible insects are a good source of bioactive compounds and have anti-inflammatory properties, mainly the inhibitory activity of the inflammation enzymes lypoxigenase (LOX) and cyclooxygenase (COX)] [61,62]. Additionally, unsaturated essential ω-3 and ω-6 fatty acids are abundant in several insects and can be increased through their feeding [61]. This is an important aspect for human health since these essential fatty acids, particularly ω-3, are known to have anti-inflammatory properties [63]. Furthermore, phytosterols (C28 or C29 sterols and stanols) are natural components of plant membranes and, despite insects, are unable to synthesize sterols from isoprenoid precursors; they obtain them from dietary sources. Their importance in preventing inflammation relies on their capability to inhibit several pro-inflammatory mediators through the inhibition of the nuclear factor kappa-light-chain enhancer of the activated B-cells (NF-kB) pathway. Other anti-inflammatory compounds found in insects are chitin and chitosan (produced by the deacetylation of chitin); it has been proven that chitin fragments of <40 µm have anti-inflammatory properties that are based in the modulation of the release of interleukin (IL)-10 and that they regulate the chronicity and intensity of local inflammation [61].

Cytokines belong to a broad family of proteins associated with inflammatory activity and an imbalance in the cytokine network leads to enhanced inflammation; therefore, cytokines are considered valuable as predictive biomarkers of disease activity. Among cytokines, tumor necrosis factor-alpha (TNF-α) is a principal cytokine, chiefly produced by macrophages, which acts as a potent inducer of other proinflammatory cytokines and chemokines, further enhancing the inflammatory response [64]. In agreement with the potential anti-inflammatory properties of edible insects, Stull et al. (2018) observed a significantly lower level of TNF- α in plasma after 14 days of the intake of a test meal with cricket powder compared to that observed after an isocaloric placebo meal [24]. Other determined cytokines and chemokines, as markers of mucosal immunity did not show significant differences, as well as acting as a marker of mucosal immunity, leading us to consider the hypothesis that the above-mentioned anti-inflammatory properties based on cytokine modulation are only enough to change principal cytokines, such as TNF-α. Other studies with healthy participants and patients with different pathologies, more insect species, other inflammation biomarkers, and longer intervention periods are still necessary.

#### 4.1.4. Microbiota

The study conducted by Stull et al. (2018) reported some interesting findings regarding the effects of insect consumption on microbiota probiotics. While they observed a decrease in the levels of Lactobacillus spp., they found increased levels of *Bifidobacterium* following the consumption of an insect meal with cricket powder [24]. *Lactobacillus* spp. is known to play a crucial role in maintaining gut health and *Bifidobacterium* is associated with various health benefits, including improved digestion and immune function [65,66]. Despite the observed decrease in *Lactobacillus* spp., the increased abundance of *Bifidobacterium* observed in this study may be indicative of a beneficial shift in the gut microbiota. The authors suggested that the decrease in *Lactobacillus* could be due to the antimicrobial activity of chitin and chitosan present in cricket powder [24]. On the other hand, an animal study where Zucker rats were fed with chitin-rich cuticles from *Tenebrio Molitor* showed an increase in both *Bifidobacterium* and *Lactobacillus* [67]. These contradictory results highlight the need to perform more human studies to understand the long-term impacts of the consumption of different types of insects on human microbiota.

SCFAs are metabolic by-products of gut bacteria fermentation and are known to have anti-inflammatory and immunomodulatory properties [68]. They also play a crucial role in maintaining gut health by providing energy for colonocytes, contributing to gut barrier function, and influencing immune regulation [69,70]. Stull et al. (2018) noted a small reduction in acetate and propionate short-chain fatty acids (SCFAs), but not in butyrate, in the group following the cricket-meal diet [24]. Again, animal models have shown contradictory results [67]; thus, further research is needed to understand the precise mechanisms underlying these effects in humans.

### 4.2. Edible Insect Allergies

A food allergy (FA) is an adverse reaction to a specific food antigen, normally harmless to the healthy population, which is mediated by immunological mechanisms and arises in individuals susceptible to that specific allergen [71]. Immune responses to foods may produce a spectrum of symptoms and disorders, from mild forms with organ localization to serious and potentially fatal forms with systemic involvement; food-allergic responses also contribute to chronic inflammatory disorders, such as eosinophilic esophagitis and atopic dermatitis [71]. FAs are becoming a major public health problem, with over 10% of the US population being likely to suffer from at least one IgE-mediated FA [71,72,73]. Most concerning is the fact that almost half of the patients with IgE-dependent FA have experienced at least one serious anaphylactic reaction, especially in childhood and adolescence; moreover, adolescents and young adults appear now to be disproportionately affected by food-induced anaphylaxis, including fatal reactions [71,72,73].

The recent introduction of edible insects in Western countries as a novel food has raised preoccupations about their safety in terms of FAs. There were several reports of anaphylaxis following an ingestion of insects by subjects without previous allergic reactions toward insects [37,74]. Therefore, a great matter of concern is cross-reactivity, which can be defined as an immune-mediated phenomenon of an IgE antibody recognizing, binding to, and inducing an immune response to similar allergenic molecules. Such events often occur between allergenic molecules in closely related species or well-preserved molecules that belong to the same protein family, have similar functions, and present in widely different species [74,75]. 

Allergic reactions to edible insects can be associated with primary sensitization, either through environmental or occupational exposures [76]. Indeed, allergic reactions subsequent to insect consumption can be associated with cross-reactivity due to the phylogenetic relationship of insects with common inhalant allergen sources, such as HDMs. Whether a primary inhalant allergy to invertebrates could lead to a secondary FA (as already described for the pollen-food syndrome) is still a matter of debate; nevertheless, co-sensitization between edible insects and HDMs has been previously proven [74,76]. In this context, it is important to note that up to 60 % of food allergies seen in older children, adolescents, and adults are associated with a HDM inhalant allergy and that HDM exposure is one of the main causes of respiratory allergies worldwide [77,78].

Regarding food allergen cross-reactivity, crustaceans, such as shrimp, crab, crawfish, and lobster, are a frequent cause of adverse food reactions in allergic individuals [79]. Moreover, allergies to shrimp and HDMs are strictly interconnected as both mites and shrimps are invertebrates and share cross-reacting allergens [78]. Concerning edible insects, adverse reactions described after their ingestion can be caused by cross-reactivity with crustaceans (as well as HDM inhalant allergens); apparently, this is mostly due to the presence of the two most common allergens among invertebrates: tropomyosin and arginine kinase [74]. 

#### 4.2.1. Tropomyosin and Arginine Kinase

Tropomyosin belongs to a family of highly conserved proteins with multiple isoforms and is found in both muscle and non-muscle cells of all species of vertebrates and invertebrates. Allergenic tropomyosins are found in invertebrates, such as crustaceans, arachnids, insects, and mollusks; whereas, vertebrate tropomyosins are nonallergenic [79]. In accordance, the allergenicity studies presented in this review (Table 2) identified tropomyosin in edible insects and identified it as a major cross-allergen. Such was true in the case of the cricket *Gryllus bimaculatus* in shrimp-allergic subjects [36], the mealworm in shrimp- and HDM-allergic subjects [35,37], and the buffalo worm, silkworm and the cricket *Gryllodes sigillatus* in subjects allergic to shrimp, HDMs or mealworms [37]. In the epidemiological study enrolled in Zimbabwe (Table 2), a frequent co-sensitization to the edible insect mopane worm (*Imbrasia belina*) and mold mite (*Tyrophagus putrescentiae*) extracts was observed [34]. The authors suggested that this might be due to reactivity to pan-allergens, such as tropomyosin [34]. Additionally, in a severe food anaphylaxis case induced by the mealworm, proteomic analysis was used to identify proteins to which the patient was sensitized, which led to the detection of tropomyosin [33].

Arginine kinase, the other major invertebrate-pan-allergen, is a protein with a highly conserved amino acid sequence among various invertebrate species. Arginine kinase is widely distributed in various insects, such as the yellow mealworm (*Tenebrio molitor*), the field cricket (*Gryllus bimaculatus*), and the house cricket (*Achaeta domesticus*), in shrimps, such as the black tiger prawn (*Penaeus monodon*), the king prawn (*Penaeus latisulcatus*), and the giant freshwater prawn (*M. rosenbergii*), and in crabs, such as the blue swimming crab (*Portunus pelagicus*) [74]. Many allergens present in dust mites are also arginine kinases [80]. In agreement, in one of the cross-reactivity studies analyzed in this review, the arginine kinase (along with tropomyosin) from the yellow mealworm was described as a major cross-reactive protein in patients allergic to crustaceans and HDM [35]. However, no mentions of arginine kinase were found in the other two cross-reactivity studies with extracts of the same insect species and other species [36,37], nor in the case report of acute anaphylaxis induced by the mealworm [33] (Table 2). In the case of crickets (*Gryllus bimaculatus*), arginine kinase was previously identified as the major allergen responsible for the binding of cricket allergens with IgE in patients with shrimp allergies [81]. Despite this, in their immunoblotting analysis, Kamemura et al. (2019) only detected the HMW tropomyosin band [36]. The authors interpreted such an inconsistency by suggesting that the allergen solution used in the study was extracted using a different method than in other studies and that the sera used from individuals with shrimp allergies were focused on food allergies and not on inhalant allergies [36]. In addition, no mentions of arginine kinase were found regarding the buffalo worm (*Alphitobius diaperinus*), mealworm larvae (*Tenebrio molitor*), cricket (*Gryllodes sigillatus*), grasshopper (*Locusta migratoria*) and silkworm larvae (*Bombyx mori*) proteins extracts [37] (Table 2). When considering the mention of arginine kinase as a major pan-allergen, additional studies on this allergen should be performed. 

#### 4.2.2. Other Allergens

Besides pan-allergens, some other proteins have been mentioned as minor allergens that belong to distinct families; they include the larval cuticle proteins (LCPs), hexamerin, α-amilase, and many others, such as apolipophorin III, the chemosensensoryprotein, the cockroach allergen-like protein, the receptor for activated protein kinase, the heat shock protein (HSP) 70, or the odorant-binding protein. These proteins have arisen as being very specific to edible insects and, despite this, are much less present in arthropods, mollusks, or nematodes; they share well-conserved amino acid sequences and highly similar quaternary structures [74,82]. 

The LCPs are a well-conserved group of proteins that play a key role in the deposition of the newly synthesized chitin chains forming the chitinous shell during the ecdysone-driven molt periods of insect larvae; they closely resemble some other pupal cuticle proteins. LCPs are abundantly represented in the yellow mealworm, most particularly as proteins A1A and A2B [33,83]. Accordingly, the study performed by Lamberti et al. (2021) (Table 2) using this insect species and the buffalo worm, the cricket Gryllodes sigillatus, the grasshopper, and the silkworm larvae led researchers to consider the LCP to be one of the most cross-reactive proteins [37]. In the reported case of food anaphylaxis induced by the mealworm, sensitization to the LCPs A1A and A2B was detected after an analysis of the patients’ sera [33] (Table 2).

Hexamerin and α-amilase are other proteins for which sensitization was detected in the patient with *Tenebrio-molitor*-induced food anaphylaxis [33] (Table 2); it is plausible that they were not detected in the other studies presented in this review because they are not pan-allergens like tropomyosin or arginine kinase. Even so, hexamerins are known to be ubiquitous in insects, with hexamerin 1B identified in *Gryllus bimaculatus* as a minor allergen in prawn-allergic patient’ sera and hexamerin-like protein 2 (HLP2) mentioned as a new allergen found in crickets [81,84]. Quite interestingly, HLP2 is referred to as being involved in both FAs and occupational allergies in factory workers who manufacture food for reptiles [84]. Similarly, α-amylase is reported as being a responsible allergen in specific cases of occupational mealworm allergies by employees working in contact with mealworms in pet stores [85]. 

#### 4.2.3. Effects of Food-Processing Technologies Used to Reduce Allergies

Some effects of food processing on edible-insect-induced allergies have been tested. In a study presented in this review, a partial reduction in cross-allergenicity via thermal processing was achieved by boiling or frying buffalo worm, mealworm larvae, the cricket *Gryllodes sigillatus*, grasshoppers, and silkworm larvae [37] (Table 2). Those results are partially in accordance with previous ones where the co-sensitization between edible insects and crustaceans was reported not to be significantly diminished by thermal treatment; although, there was an impact on the intensity and types of allergens that were detected [76]. 

Another method was presented via the study of Verhoeckx et al. (2014) [35] involving *Tenebrio molitor* protein extracts in which the stability was tested using a static pepsin digestion model; it was shown that cross-reactive yellow mealworm proteins were moderately stable (Table 2). In other studies, in vitro digestion has been shown not to eliminate the IgE-binding capacity of mealworm tropomyosin; meanwhile, in others, the heat and proteolysis stability of tropomyosin from the mud crab (*Scylla serrata*) and the tropical oyster (*Crassostrea belcheri*) have been similarly pointed out [76,80]. Additional methods using the microwave-assisted enzymatic hydrolysis of tropomyosin achieved a degree of hydrolysis greater than 50%. However, it can be determined that in general, insect tropomyosin seems to be able to maintain its allergenicity even after most thermal or enzymatic treatments and the heat resistance of the major allergens of edible insects implies that both cooked insects and insect protein-containing food products retain some intact allergenicity [76,80].

For now, according to current European food legislation on novel foods (EU Reg. 2015/2283), insects intended as an alternative source of food proteins for human consumption are considered novel foods. Since food allergens are mostly proteins, greater efforts in the analysis and identification of the potential allergenicity of these novel proteins should be a fundamental activity to ensure a high level of food safety for European consumers. Additionally, including allergenicity assessments as part of the risk assessment of novel food is crucial; they should be carried to the existing allergic population identified by the immunoglobulin E (IgE) cross-reactivity [86].

## 5. Conclusions

The results of the studies reviewed contribute to our understanding of the nutritional and health implications of consuming insect protein. Insect protein ingestion leads to a higher area under the curve (AUC) for EAA and leucine compared to beef protein; it is similar to milk and soy protein. This suggests that insect protein can be a suitable alternative used to promote muscle protein synthesis. The presence of chitin in insect-based products may result in a slower digestion rate and a delayed release of the amino acids. Furthermore, the absorption of certain micronutrients, such as iron, may be dependent on the chitin content of the insect species. Regarding metabolic and inflammatory markers, glucose levels did not differ but insulin levels were significantly lower after consuming insect-based products compared to other protein sources. Inflammation markers showed mixed results, with lower levels of tumor necrosis factor observed after cricket consumption. While the reviewed studies provide promising insights into the potential metabolic and anti-inflammatory benefits of consuming edible insects, further research is needed. No significant differences were observed in terms of the desire to eat and the prospective food consumption between insect protein and other protein sources. The impact of insect protein on appetite regulation may be influenced by the specific amino acid composition; more data on the amino acid content of different insect species are needed to fully assess their value in comparison to other protein sources. On the other hand, a significant concern regarding the consumption of edible insects is cross-reactivity, which was observed between insect allergens and known allergens, such as shrimp and house dust mites. Tropomyosin and arginine kinase were two major pan-allergens found in invertebrates; they are associated with allergic reactions to edible insects, along with other minor insects allergens, such as larval cuticle proteins, hexamerin, α-amylase, and several other proteins. Food processing techniques can partially reduce cross-allergenicity in edible insects but the impact may vary. Further studies are certainly needed to explore these allergens and develop effective processing methods to reduce allergies. 

The strength of this systematic review was its contribution to our understanding of the effects of insect consumption on diverse aspects of human health based on human interventions, such as nutrient absorption, muscle protein synthesis, satiety, microbiota, metabolic, and inflammatory biomarkers. However, limitations existed, such as the reduced number of studies, the lack of participants in some cases, and the sparseness of the available results; due to frequently involving different insect species, creating comparisons was a hard task. More insect species should be investigated, along with a broader range of biomarkers and longer intervention periods, to gain a better understanding of the comprehensive effects of insect consumption on human health.

## Figures and Tables

**Table 1:** Effect of edible insect consumption on human health and characterization of randomized controlled trials (RCT).

Author, Year, (Country)	Type of RCT	Test	Control	Participants	Intervention	Outcomes Data Collection	Results
Iron Absorption							
Mwangi et al., 2022 (Netherlands) [31]	Crossover, acute (1 day)	800–900 g porridge meal with low phytate or high phytate maize flour + 100 g of [57Fe]-labeled ground Cr or 50 g of unlabeled ground Cr.	Placebo = 800–900 g porridge meal with low phytate or high phytate maize flour. Fe content matched by the addition of FeSO4	20 Females, iron-depleted, mean age 24.7 ± 2.9 years; mean BMI 21.6 ± 1.9 kg/m^2^	Administration of different meals on consecutive days, after overnight fast, with a 14-day interval between insect and placebo meals. Meals ingested in two portions of 400–450 g each, served at breakfast and ≥3 h after. Lunch is provided after the second serving.	Hemoglobin, serum ferritin, STR, fractional iron absorption. Blood samples collected on days 0, 23, and 39 after the ingestion.	↔ Hemoglobin ↔ Serum ferritin. ↑ STR for insect compared to placebo. ↔ Fractional iron absorption in high phytate meals. ↓ Fractional iron absorption for low phytate meal with labeled or unlabelled insect, compared to low phytate meal placebo.
Amino acid Absorption and muscle protein synthesis							
Hermans et al., 2021 (Netherlands) [28]	Parallel, acute (1 day)	64 g of Lw protein powder in 300 mL water (30 g protein, 312 kcal).	Alternate protein source: 40 g of dried milk protein concentrate in 300 mL water (30 g protein, 142 kcal)	24 Males, healthy, mean age 23.0 ± 3.0 years; BMI 23.1 ± 2.7 Kg/m^2^	Exercise (one leg exercised and one leg at rest) after overnight fast, followed by administration of insect or milk protein.	Plasma amino acids (EAA, NEAA, TAA, Leu, Phe, Tyr), glucose, insulin, muscle protein synthesis rate. Blood samples collected before and after exercise, and at postprandial states (20 to 300 min); muscle biopsy collected for rest and exercised leg after exercise, and 120, 300 min after ingestion of insect or control.	↔ Glucose and insulin. ↓ Peak Leu, Phe, EAA, NEAA, TAA for insect compared to milk protein. ↑Peak Tyr for insect, compared to milk protein. ↔ AUC EAA, NEAA and TAA. Amino acid plasma levels peaked at 30 min for milk and at 60 min for insect. ↔ Protein synthesis rate at rest and after exercise.
Vangsoe et al., 2018 (Denmark) [25]	Crossover, Acute (1 day)	30.5 g of protein isolate from Lw (25 g protein) in 400 mL water (100 Kcal).	Placebo = 400 mL of water Alternative protein sources: C1 = 25 g of soy protein isolate in 400 mL of water C2 = 25 g of whey protein isolate in 400 mL of water	Six Males, healthy, mean age 24 ± 1 years.	Administration of products on four different days, after overnight fast, with one-day intervals between each intervention. Ingestion of insect, soy, whey, and placebo products within 1 min.	Plasma amino acids (EAA, BCAA, Leu) and insulin. Blood samples collected at pre-prandial (0 min) and postprandial states (20 to 120 min).	↑ AUC of EAA, BCAA, and Leu for all protein sources compared to placebo. ↑ AUC of EAA, BCAA, and Leu for whey, compared to soy and insect. Amino acid plasma levels peaked at 60 min for whey and soy and at 120 min for insect. ↓ Insulin at 20 and 40 min for insect compared to whey and soy.
Vangsoe et al., 2018 (Denmark) [26]	Parallel, chronic (8 weeks)	Protein bar of banana, ginger, and oats, supplemented with 0.4 g of Lw protein/kg body weight (137 kcal).	Placebo = Isocaloric bar with no insect protein (3.6 g protein).	18 Males, healthy, mean age 24.2 ± 2.6 years; body weight 79.9 ± 9.0 kg, height 186.6 ± 6.6 cm	Two insect or placebo bars per day: one bar 1h after resistance training and one bar 1 h before sleep on training days, 4 days a week.	Body composition (BW, FM, BMC, FBFM), muscle strength (1 RM leg and bench press), energy, and macronutrient intake. Body composition (DXA) and muscle strength measured 1 week before the start and 2 days after the last training session; 3-day dietary records before and during the intervention.	↔ BW, FM, FBFM, and strength. ↑ Protein intake for insect compared to placebo (2.3 g/kg/day for test, 1.7 g/kg/day for control) ↓ CHO intake for insect compared to placebo (4.8 g/kg/day, for test, 5.8 g/kg/day for control) ↔ Energy and fat intake
Amino acid Absorption and Appetite Regulation							
Dai et al., 2022 (Canada) [29]	Crossover, acute	400 mL beverage with 25 g of Cr-derived protein.	Alternative protein source: 400 mL beverage with 25 g of beef-derived protein	20 Males, healthy, mean age 23 ± 4 years.	Administration of beverages after overnight fast, with a 7-day interval between insect and beef. Beverages drank in 5 min; ad libitum meal after 300 min.	Plasma amino acids (BCAA, EAA, NEAA, TAA, Leu), glucose, insulin, perceived hunger, fullness, desire to eat, PFC, satiety hormones (GLP-1, PYY). Self-filled rating questionnaires (VAS) and blood samples, before ingestion and postprandial every 15 min, until 300 min.	↔ Glucose, GLP-1, and PYY. ↓ Insulin for insect. ↑ AUC of Leu, BCAA and EAA for insect ↓ AUC of NEAA and TAA for insect. Amino acid plasma levels peaked at approx 60 to 80 min for beef and at approx 90 to 100 min for insect. ↓ Hunger for beef. ↔ Fullness, desire to eat, and PFC. ↔ Energy intake after ad libitum meal.
Miguéns-Gómez et al., 2022 (Spain) [30]	Crossover, acute (1 day)	170 g Cocoa milkshake with 25 g of Lw powder (20 g protein; 194.1 kcal).	Placebo = 145 g Cocoa milkshake (5.7 g protein; 66.7 kcal) Alternative protein source: 205 g Cocoa milkshake with 60 g almond flour (20.1 g protein; 443.2 kcal)	12 Females, 17 Males, healthy, 22 to 33 years, BMI < 40 kg/m^2^	Administration of a preload of insect, almond, and placebo on separate days, after overnight fasting, with 6–7-day intervals; 1 h and 4 h after preload, ad libitum breakfast and lunch.	Subjective desire to eat, PFC, feelings of indigestion, energy and protein intake after ad libitum meal. Self-filled rating questionnaires (VAS) before ingestion and every 1 h after, until 8 h.	↓Desire to eat, for insect and almond compared to placebo, 1 h after preload. ↓PFC, for insect and almond compared to placebo, 1 h and 5 h after preload. ↔ Desire to eat and PFC for insect compared to almond. ↔ Cumulative energy and protein intake (breakfast + lunch). ↑ Sensation of indigestion for insect, compared to almond and placebo.
Skotnicka et al., 2022 (Poland) [32]	Crossover, acute (1 day)	240 kcal pancake of wheat flour, egg and milk + 10%, 20% or 30% flour of Tm, Cr, or Lw. Total protein (g) of each pancake was: Tm, 8.2, 9.3, 10.2; Cr, 9.6, 12.0, 14.4; Lw, 9.6, 11, 12.6.	Placebo = 240 kcal pancake (7.6g protein from milk and egg).	41 Females, 33 Males, healthy, 20 to 28 years, BMI 18.5 to 25 kg/m^2^	Administration of insect or placebo products on separate days, after overnight fasting, with 1-day intervals (eight samples in total). Pancakes are eaten within 5 min.	Subjective feelings of hunger and satiety. Self-filled subjective rating questionnaires (VAS) before and after ingestion at 30-minute intervals for 180 min. Pearson correlation coefficients determined for satiety levels and physico-chemical parameters of products.	↔ Hunger for 10%, 20%, 30% Tm and Cr, 10% Lw, placebo between men and women. ↑ Hunger for 20% and 30% Lw in women compared to men. ↓ AUC of Hunger for 20% and 30% Cr, and 30% Lw. ↔ Satiety for 10% and 30% Tm,10, 20 and 20% Cr, 10% Lw, and placebo between men and women. ↑ Satiety for 20% Tm, 20% and 30% Lw in women compared to men. ↑ AUC of satiety for 30% Lw, 20% and 30% Cr. Physico-chemical characteristics related to induction of satiety were: protein (positive relation) > carbohydrates, water (negative relation) > dietary fiber (positive relation).
Microbiota							
Stull, et al., 2018 (USA) [24]	Crossover, Chronic (14 days)	Breakfast meal with Cr powder: muffins (15g Cr) and shake (10 g Cr), 21.7 g protein; 569.3 kcal.	Breakfast meal: pumpkin muffins and a chocolate shake (9.3 g protein, 495.3 kcal).	Eleven Females, nine Males, healthy, mean age 26.4 ± 6.3; mean BMI 23.4 ± 2.5	Administration of insect or placebo products after overnight fasting in separate weeks, with 14-day washout intervals.	Plasma glucose, Na, K, CO_2_, Cl, Ca, urea nitrogen, creatine, ALP, ALT, AST, bilirubin, albumin, total protein; Systemic inflammation: plasma cytokines and chemokines; Mucosal immunity: fecal sIgA; Microbial metabolism: fecal SCFAs and bile acids; Microbiota; GI function: feelings related to digestive health. Collection of blood and stool after overnight fast, before intervention, after insect and control interventions.	Blood chemistry: ↔ Plasma Na, K, Cl, CO_2_, Ca, glucose, urea nitrogen, creatine, ALP, ALT, AST, bilirubin, albumin, total protein Inflammation: ↓ TNF-α for insect, ↔ for all other cytokines and chemokines Mucosal immunity: ↔ sIgA Microbial metabolism: ↓ SCFA acetate and propionate for insect; ↔ Bile acids Microbiota: ↓ Probiotic *Lactobacillus* spp. and *Leuconostoc;* ↑ Probiotic *Bifidobacterium animalis*, for insect ↔ GI function.
Disease treatment							
Hu, et al., 2020 (China) [27]	Parallel, chronic (3 months)	Routine medication (β2 receptor agonist and inhaled glucocorticoids) + 30 g compound Caoshi silkworm Granules + 5 g Astragalus in granules.	Routine medication	Test: twelve Males, eight Females Control: eight Males, twelve Females; COPD, mean age 64.9 ± 8.2; mean BMI 21.3 ± 4.0	Daily ingestion of medication or medication + granules.	Respiratory symptoms, activity and impact (SGRQ), lung function (FEV1, FVC, FEV1/FVC). Data collected before intervention and at the end of month 3.	↓ SGRQ scores on symptoms, activity, and impact for insect. ↔ Pulmonary function 3 months after the intervention.

Cr, Cricket (*Acheta domesticus*, *Gryllodes sigillatus*); Lw, Lesser mealworm (*Alphitobius diaperinus*); Tm, Mealworm (*Tenebrio molitor*). Iron and Amino acids: STR, Soluble transferrin receptor. EAA, essential amino acids; NEAA, non-essential amino acids; BCAA, branch chain amino acids; TAA, total amino acids; Leu, leucin; Phe, phenylalanine; Tyr, Tyrosine; AUC, area under the curve. Body composition: DXA, Dual-energy X-ray absorptiometry; BW, body weight; FM, fat mass; BMC, bone mineral content; FBFM, Fat- and bone-free mass. Appetite: VAS, with visual analog scales for subjective rating; PFC, prospective food consumption; GLP-1, glucagon-like peptide; PYY, peptide PYY. Blood chemistry: ALP, alkaline phosphatase, ALT, alanine aminotransferase, AST, aspartate aminotransferase. Intestinal antibodies and metabolism: sIgA, secretory intestinal epithelial immunoglobulin A; SCFA, short chain fatty acids. COPD, Chronic obstructive pulmonary disease. SGRQ, St George’s Respiratory Questionnaire. FRV1, forced expiratory volume-one second, FVC, forced vital capacity, FEV1/FVC, forced expiratory volume-one second/FVC. ↔ No significant differences between the test and control; ↑ significant increase or higher levels; ↓ significant decrease or lower levels.

**Table 2:** Effect of edible insect consumption on human health–characterization of studies on allergies.

Author, Year, (Country)	Type	Test/Case	Control	Participants/Case	Intervention/Event	Outcomes Data Collection	Results
Cross-reactivity studies with patients’ sera							
Lamberti et al., 2021 (Italy) [37]	Specific IgE to allergen extracts	Thermal processing of buffalo worm (*Alphitobius diaperinus*), mealworm larvae (*Tenebrio molitor*), cricket (*Gryllodes sigillatus*), grasshopper (*Locusta migratoria*), and silkworm larvae (*Bombyx mori*) proteins extracts.	Protein extracts from the mentioned raw insects.	Patients allergic to: house dust mites (HDM) (n = 28); shrimp (n = 8); mealworm (n = 2); Control patients: not allergic to either shrimps or HDM (n = 3); all adults.	Patients’ sera exposure to the proteins extracted from the insects, with or without thermal processing.	Insects’ protein profiles after each thermal treatment; screening in patients’ sera for the immunorecognition of the extracted insects’ proteins.	71% of HDM and 87% of shrimp allergic patients recognizing at least one insect protein extract; tropomyosin and larval cuticle protein (LCP) as the most cross-reactive proteins; partial reduction of cross-allergenicity by thermal processing.
Kamemura et al., 2019 (Japan) [36]	Specific IgE to allergen extracts	*Gryllus bimaculatus* (cricket) allergen extracts.	Patients without shrimp allergy (n = 6).	Patients allergic to shrimp (n = 9).	Patients’ sera exposure to Gryllus and shrimp allergens extracts.	Identification of allergenic proteins in Gryllus and shrimp; estimation of allergen-specific IgE levels for shrimp- and Gryllus in patients sera.	Strong correlation between shrimp- and Gryllus-specific IgE responses; tropomyosin as the major allergen in shrimp and Gryllus.
Verhoeckx et al., 2014 (Netherlands) [35]	Specific IgE to allergen extracts; Basophil activation test	Yellow mealworm (*Tenebrio molitor*) protein extracts.	Patients allergic to grass pollen, peanuts, fish, or eggs and/or milk; also not allergic to crustaceans or HDM (n = 15).	Patients allergic to crustaceans and HDM (n = 7).	Patients’ sera exposure to different yellow mealworm protein fractions.	Identification of cross-reactive proteins of the yellow mealworm; indirect basophil activation.	IgE from HDM- and crustacean allergic patients cross-reaction with yellow mealworm proteins; induction of basophil activation; tropomyosin and arginine kinase as major cross-reactive proteins; moderate stability of cross-allergens after static pepsin digestion model test.
Epidemiological study							
Ndlovu et al., 2021 (Zimbabwe) [34]	RCT Parallel, acute (1 day)	In-house preparation of mopane worm (*Imbrasia belina*) inhalant allergen extracts and other 10 inhalant allergen extracts locally relevant.	No control.	Patients ≥ 10 years old (total of 29 households) from a mopane worm harvesting rural community (n = 17, 13 females and 4 males).	Exposure by skin prick to the allergens.	Allergen sensitization patterns assessed by skin prick test, lung function (spirometry), and fractional exhaled nitric oxide levels (allergic airway inflammation).	Prevalence of sensitization to *Imbrasia belina* of 50%; respiratory health symptoms amongst participants sensitized to mopane worm; prevalence from 22 to 72% for other allergens including cockroach, mosquito, and HDM.
Case report							
Beaumont et al., 2019 (France) [33]	Case study	Severe food anaphylaxis induced by mealworm (*Tenebrio molitor*).	Not applicable.	Patients allergic to HDM but not to crustaceans; 31 years old.	Consumption of 1 cooked larvae, probably fried.	Prick-tests; serum proteomic analysis (identification of *T. molitor* proteins to which he was sensitized).	Sensitization to hexamerin, tropomyosin epitopes, α-amylase (identified as an allergen in mealworms and with structural homology with HDM), and to larva cuticle proteins A1A and A2B (known *T. molitor* allergens).

## Data Availability

No new data were created or analyzed in this study. Data sharing is not applicable to this article.

## Supplementary Materials

The following supporting information can be downloaded at: https://www.mdpi.com/article/10.3390/nu15143076/s1, Table S1: Risk of bias assessment.
