# Supplementary material for: Effects of Insect Consumption on Human Health: A Systematic Review of Human Studies
Source: Nutrients. 2023 Jul 8;15(14):3076. doi: 10.3390/nu15143076 (PMC10386438; doi:10.3390/nu15143076)
Supplement: Supplementary file 1 [file nutrients-15-03076-s001.zip › nutrients-2482247-supplementary.pdf]

Table S1. Risk of bias assessment

Domains	Randomization process [1]	Deviations from intended intervention [2]	Carryover effects [3]	Missing outcome data [4]	Measurement of outcome [5]	Overall risk of bias
Signaling questions	Concealed allocation sequence / Random allocation sequence	Blinded to participants / Blinded to investigators / compliance check /	Washout time appropriate for disappearance of carryover effects	Flow of participants described / outcome data for all participants that finished the protocol	Appropriate method to measure outcome / potential influence by knowledge of intervention or mismatch of test and control products	Low risk of bias/Some concerns/High risk of bias
Mwangi et al., 2022 (Netherlands) [31]	NI/Y Concerns	Y/N/Y Concerns	PY Low risk	Y/Y Low risk	Y/PN Low risk	High risk
Hermans et al., 2021 (Netherlands) [28]	PY/Y Low risk	Y/Y/Y Low risk	NA	Y/Y Low risk	Y/N Low risk	Low risk
Vangsoe et al. 2018a (Denmark) [25]	NI/NI High risk	Y/NI/PY Concerns	PY Low risk	Y/Y Low risk	Y/PN Low risk	High risk
Vangsoe et al. 2018b (Denmark) [26]	NI/NI High risk	Y/N/Y Concerns	NA	Y/Y Low risk	Y/PY Concerns	High risk
Dai et al., 2022 (Canada) [29]	Y/Y Low risk	Y/Y/Y Low risk	PY Low risk	Y/Y Low risk	Y/N (except for one outcome that was participant-assessed) Low risk	Low risk
Miguéns-Gómez et al., 2022 (Spain) [30]	NI/NI High risk	Y/N/Y Concerns	PY Low risk	N/NI High risk	Y/PY Concerns	High risk
Skotnicka et al., 2022 (Poland) [32]	NI/NI High risk	Y/Y/NI Concerns	PY Low risk	N/Y Concerns	Y/PY Concerns	High risk
Stull, et al., 2018 (USA) [24]	Y/NI Low risk	Y/Y/Y Low risk	PY Low risk	Y/Y Low risk	Y/PY Concerns	Some concerns
Hu, et al., 2020 (China) [27]	NI/NI High risk	N/N/Y Concerns	NA	Y/Y Low risk	Y/PY Concerns	High risk

Answers to signaling questions: Y = yes; PY = probably yes; N = no; PN = probably no; NI = no sufficient information; NA = not applied.
1 Assessment of risk of bias from randomization process - low risk: Y or PY for both questions OR Y or PY to allocation concealment AND NI on random allocation sequence as long as there is no imbalance on the baseline of both intervention groups; concerns: NI for sequence allocation concealment, irrespective of

random allocation sequence, or Y/PY for sequence allocation concealment AND N or PN for random allocation sequence; high risk: N or PN for sequence allocation concealment irrespective of random allocation sequence OR NI for both questions.

2 Assessment of risk of bias due to deviations from intended intervention - low risk: Y or PY for all signaling questions; concerns: at least one N or PN or NI; high risk: N or PN or NI for all signaling questions.

3 Assessment of risk of bias due to carryover effects in crossover interventions - low risk: Y or PY; concerns: NI; high risk: N or PN.

4 Assessment of risk of bias due to missing outcome data - low risk: Y or PY for all signaling questions; concerns: one Y or PY, AND one NI or N or PN; high risk N or PN or NI for all signaling questions.

5 Assessment of risk of bias due to outcome assessment - low risk: Y or PY for appropriate method of measurement AND N or PN for potential influence; concerns: Y or PY for appropriate method of measurement AND NI or Y or PY for potential (for participant-reported outcomes, the assessment of outcome is always considered as potentially influenced by knowledge of intervention, independently of blinding); high risk: NI or N or PN for appropriate method of measurement, irrespective of the other signaling question.

Overall evaluation: Low risk of bias, when all assessed domains are low risk; Some concerns, if any domain was assessed as some concerns AND no domain was assessed as high risk; High risk of bias, when at least one domain was assessed as high risk, or multiple domains were assessed as some concerns [24].
